# Analysis of the Use of Over-the-Counter Therapy for the Prevention and Treatment of COVID-19

**DOI:** 10.3390/medicina61050803

**Published:** 2025-04-25

**Authors:** Ivan Vukosavljević, Nataša Djorić, Ivana Vukosavljević, Jasmina Milovanović, Nataša Zdravković, Katarina Djordjević, Nebojša Zdravković, Marina Kostić, Ana Barjaktarević, Snezana Cupara, Ivan Čekerevac, Nevena Vasović, Aleksandra Tomić Lučić, Marija Šorak, Nikola Mirković, Olivera Kostić

**Affiliations:** 1 Faculty of Medical Sciences, University of Kragujevac, 34000 Kragujevac, Serbia; ivanvukosavljevic2@gmail.com (I.V.); natasa.djoric@gmail.com (N.D.); ivana.vukosavljevic035@gmail.com (I.V.); 2 Department of Pharmacology and Toxicology, Faculty of Medical Sciences, University of Kragujevac, 34000 Kragujevac, Serbia; jasminamilo@yahoo.com (J.M.); marrina2006kg@yahoo.com (M.K.); 3 Department of Internal Medicine, Faculty of Medical Sciences, University of Kragujevac, 34000 Kragujevac, Serbia; natasasilvester@gmail.com (N.Z.); icekerevac@gmail.com (I.Č.); atomiclucic@gmail.com (A.T.L.); 4 Clinic of Gastroenterology and Hepatology, Serbia University Clinical Center of Kragujevac, 34000 Kragujevac, Serbia; 5 Center for Research on Harmful Effects of Biological and Chemical Hazards, Faculty of Medical Sciences, University of Kragujevac, 34000 Kragujevac, Serbia; nzdravkovic@fmn.kg.ac.rs (N.Z.); olivera.milovanovic09@gmail.com (O.K.); 6 Department of Pharmacy, Faculty of Medical Sciences, University of Kragujevac, 34000 Kragujevac, Serbia; kacka96@gmail.com; 7 Department of Biomedical Statistics and Informatics, Faculty of Medical Sciences, University of Kragujevac, 34000 Kragujevac, Serbia; 8 Pharmacy Department, Faculty of Medical Sciences, University of Kragujevac, 34000 Kragujevac, Serbia; ana.radovanovickg@gmail.com (A.B.); snezanacupara@yahoo.com (S.C.); 9 Faculty of Hotel Management and Tourism, University of Kragujevac, 36210 Vrnjačka Banja, Serbia; nevena.vasovic@kg.ac.rs; 10 Clinic for Rheumatology and Allergology, University Clinical Center Kragujevac, 34000 Kragujevac, Serbia; 11 Department of Gynecology and Obstetrics, Faculty of Medical Sciences, University of Kragujevac, 34000 Kragujevac, Serbia; 12 Department of Biomedically Assisted Fertilization, Clinic of Gynecology and Obstetrics, University Clinical Center Kragujevac, 34000 Kragujevac, Serbia; 13 Vascular Surgery Center, University Clinical Center Kragujevac, 34000 Kragujevac, Serbia; drnikolamirkovic@gmail.com; 14 Department of Surgery, Faculty of Medical Science, University of Kragujevac, 34000 Kragujevac, Serbia

**Keywords:** self-medication, COVID-19, socio-demographic characteristics, vitamins, supplements

## Abstract

*Background and Objectives*: Self-medication includes the use of drugs or herbal preparations based on one’s own discretion or the recommendation of another person, often a family member, friend, neighbor, or even a pharmacist, without prior examination and consultation with a doctor. The goal of this study was to determine respondents’ reasons for using self-medication, determine the frequency of self-medication, and analyze differences in respondents’ attitudes about self-medication in relation to several factors. *Materials and Methods*: The study was conducted as a cross-sectional observational study. The respondents were patients seeking medical examination at the Health Center in Jagodina who had used over-the-counter medications during the COVID-19 pandemic. A total of 175 respondents participated in the study. The study design provided answers to questions about the respondents’ self-medication habits during the COVID-19 pandemic. *Results*: More than half of the respondents (53.71%) bought medicines without a prescription, with most cases involving analgesics (52.83%). Almost three-quarters of the respondents were completely vaccinated (74.29%) against COVID-19. Additionally, 39.62% of participants used vitamins as part of their self-medication during the COVID-19 pandemic. Among the vitamins, respondents most commonly used a combination of vitamins C and D (20.75%), vitamin D (5.66%), vitamin C (5.66%), and vitamin A (1.89%). *Conclusions*: Self-medication for the treatment of coronavirus is more often used by younger respondents, who are not yet married, do not have their own income, and rarely visit a doctor. As for supplements, respondents used zinc, and as for vitamins, respondents mostly used a combination of vitamins C and D.

## 1. Introduction

Self-medication is an important segment of self-care that refers to taking medicines without a prescription, abusing old prescriptions, sharing medicines with family members or friends, and using medicines that have been left unused [1]. Self-medication is defined as taking medication on one’s own initiative without formal medical training or based on the recommendation of some health workers (physicians, pharmacists, or others). Self-medication also includes the use of drugs or herbal preparations based on one’s own discretion or the recommendation of another person, often a family member, friend, neighbor, or even a pharmacist or doctor [2].

The decision to use self-medication can be influenced by various factors, including individual, economic, and cultural ones [3]. The reasons why people decide to self-medicate are limited access to health care or its unavailability, distance from health institutions, lack of time, misconceptions about the knowledge of doctors but also the urgency of treatment, beliefs that enough is known about symptoms and diseases, etc. [4]. Self-medication is a global phenomenon whose prevalence in most parts of the world ranges from 32.5% to 81.5%; in some regions, it exceeds 90%, which is directly related to the development of countries and the availability of medicines. Self-medication is more common in developing countries compared to countries with low and medium income levels [5].

The most commonly used drugs in the process of self-medication are analgesics, antipyretics, and antibiotics [3]. Over-the-counter medicines are most often taken for coughs and colds, headaches, muscle and bone pain, and gastrointestinal disorders [1]. These drugs are often advertised in the media as safe to use. This practically means that the patient assumes responsibility for their health condition, as well as for the consequences of taking medication without medical supervision [6]. With the development of the health care system, it is expected that this type of treatment for patients with milder health problems will become dominant [7]. The main role in this process will be played by pharmacists, who are the primary source of information for patients.

During the coronavirus pandemic, the self-medication of the population came to the fore due to the fear of disease, limited access to health care, and misinformation published on social networks [8]. The prevalence of self-medication for the prevention of coronavirus was indicated in 33.9% of hospitalized patients who tested positive and in the range of 4% to 88% in the general population [9].

With the beginning of the pandemic, many recommendations appeared in relation to medicine use for the prevention and treatment of COVID-19. Since these were often not official therapies, their use was part of self-medication. Among the proposed medicines were antimalarials, including hydroxychloroquine and chloroquine [10], chlorine dioxide solution, antiparasitic drugs such as ivermectin, antibiotics including azithromycin, doxycillin, and penicillin, non-steroidal anti-inflammatory drugs such as ibuprofen [11], antiviral drugs lopinavir and ritonavir, vitamin supplements such as vitamins C and D, zinc, and calcium, etc. [12].

The use of hydroxychloroquine initially attracted the attention of researchers because they obtained good results in in vitro studies and small uncontrolled studies [9]. Later clinical research on hospitalized patients did not confirm the obtained results [13]. Similar results were found in research on azithromycin [10]. There is also insufficient evidence for the effectiveness of ivermectin [14] and vitamin supplements [15]. The use of ivermectin for the prevention and treatment of COVID-19 was controversial in the scientific community due to its serious adverse effects and unclear therapeutic potential.

The rapid emergence of a vaccine against the coronavirus reduced the need for self-medication with unproven medicines [16].

An additional alarming fact about self-medication is the potential interaction of OTC products with chronic therapy. The outcome of these reactions can often be the worsening of the underlying condition or the development of new complications, which require different therapeutic approaches, further burdening the limited budget of the healthcare sector.

The goal of this study was to determine respondents’ reasons for using self-medication, determine the frequency of self-medication, and analyze differences in respondents’ attitudes about self-medication in relation to their socio-demographic characteristics, average monthly earnings, health condition, and attitude toward their chosen doctor and pharmacist during the COVID-19 pandemic.

## 2. Materials and Methods

### 2.1. Study Design and Setting

The study was conducted as a cross-sectional observational study. A total of 175 respondents participated in the study. Participants were selected based on convenience sampling. The study included adult respondents who used over-the-counter medications during the COVID-19 pandemic. The research lasted from April to July 2023.

### 2.2. Participants and Criteria

The study included adult respondents who used over-the-counter medications during the COVID-19 pandemic. The respondents were patients seeking medical examination at the Health Center in Jagodina, Serbia. Data from respondents with formal medical education were not considered.

### 2.3. Sample Size and Sampling Method

Using the statistical program G*Power 3.1 for the Student’s *T*-test, with an accepted type one error probability of α = 0.05 and a study power of 0.95, the total sample size was estimated to be a minimum of 172 respondents. The sample size was calculated based on data from a study with a similar design, considering the frequency of self-medication during COVID-19 infection: 29.6% for females and 20.2% for males [17].

### 2.4. Study Instruments

The instrument used in the research was a questionnaire consisting of three parts. The first part of the questionnaire focused on the socio-demographic characteristics of the respondents. The second part addressed respondents’ health status and their habits regarding the use of over-the-counter medications. The third part of the questionnaire examined the application of self-medication among the respondents during the COVID-19 pandemic. For the section of the questionnaire related to the respondents’ attitudes toward certain statements, responses were measured using a five-point Likert scale, with responses ranging from 1—strongly disagree to 5—strongly agree. The questionnaire items demonstrated good internal consistency, as the Cronbach’s alpha value was 0.903, which is greater than 0.7. The contents of the questionnaire proved valid and reliable, with a high degree of internal consistency.

### 2.5. Study Variables

The variables in the first section pertained to the respondents’ general socio-demographic characteristics, including gender, age, level of education, marital status, and monthly income. The second section focused on the respondents’ health status, covering the presence of acute and chronic diseases, a history of COVID-19 infection, and trust in their primary care physician and pharmacist. The third section examined the process of self-medication during the COVID-19 pandemic, while an additional part of this section explored the reasons behind the respondents’ decision to engage in self-medication during the pandemic.

### 2.6. Data Collection

The study population was formed using a convenience sampling method. All patients who visited the Health Center in Jagodina for a medical examination and met the inclusion criteria constituted the study sample. The inclusion criteria were: age over 18 and receiving healthcare services at the Health Center in Jagodina. The exclusion criteria were: age below 18, age over 85, pregnancy and lactation, the presence of malignancies, cognitive dysfunction, individuals who did not provide written consent to participate in the study, and individuals who, for any objective reason, were unable to participate in the study.

Before the research began, written approval was obtained from the Ethics Committee of the Health Center in Jagodina to conduct the survey within its facilities. After receiving approval, the survey was conducted among the participants. Participants were included in the study after signing an informed consent form, with full disclosure of information. Researchers were obligated to provide participants with a printed document outlining the purpose and objectives of the study, their rights as participants, and details on where and how they could submit complaints if they believed their rights had been violated in any way.

Participants were included successively from the first day of the study until the required total number was reached, in accordance with the sample size calculation.

The ethical standards of the study adhered to international regulations (Helsinki Declaration) and the specific legislation of the Republic of Serbia. To ensure the privacy of research subjects and the confidentiality of information, all necessary steps were taken in accordance with the Personal Data Protection Act, the Official Statistics Act, and the European Parliament Directive on personal data protection (Directive 95/46/EC).

### 2.7. Statistical Analysis

The statistical analysis of the data was performed using IBM SPSS Statistics v.23. To assess the normality of the data distribution, the Kolmogorov–Smirnov test for normality was applied. To analyze the mean values of continuous variables in relation to categorical variables with two responses, the Mann–Whitney U test was used, while the Kruskal–Wallis test was applied depending on the number of categories.

To analyze the influence of other variables on the decision to self-medicate during the COVID-19 pandemic, binary logistic regression was used. This regression determined the effect of each individual variable, as well as which variable had the strongest unique impact on the respondents’ decision to self-medicate.

Results were considered statistically significant if the *p*-value (*p*) was less than or equal to 0.05.

## 3. Results

The demographic and social characteristics of the respondents are presented in Table 1.

The respondents were between 20 and 85 years old, with an average age of 52.37 ± 14.78 years. There were more female respondents in the sample, accounting for 61.71%. About half of the sample (49.71%) consisted of respondents who had completed primary and secondary school. In terms of employment status, 59.43% of respondents were employed, while 25.71% were retired. Most of the respondents were married (69.71%) or in a relationship with a partner (14.86%). The respondents predominantly had personal monthly incomes (85.14%), while the monthly family incomes of half of the respondents (50.29%) ranged from RSD 40,000 to 80,000 (approximately EUR 350 to 680). Most of the respondents were from urban areas (80.0%). About a third of respondents (35.43%) had family members with formal medical education. The majority of respondents (84.0%) knew someone who died as a result of the COVID-19 pandemic.

Descriptive statistical analysis related to the health status of the respondents was performed using absolute and relative frequencies and is shown in Table 2.

Of the respondents who participated in this study, only 16.57% visited a doctor at least once a month. Almost half of the respondents (47.43%) had one of the chronic diseases, and in most cases (62.65%) it was hypertension. Respondents showed a high level of trust in their physician (96%) and pharmacist (92.6%). More than half of the respondents (53.71%) bought medicines without a prescription, and in most cases, they were analgesics (52.83%). More than half of the respondents tested positive for COVID-19 (60.57%), mostly only once (72.64%), and were treated in health centers (85.85%). Almost three-quarters of respondents were vaccinated (74.29%) against COVID-19.

**Figure 1:**
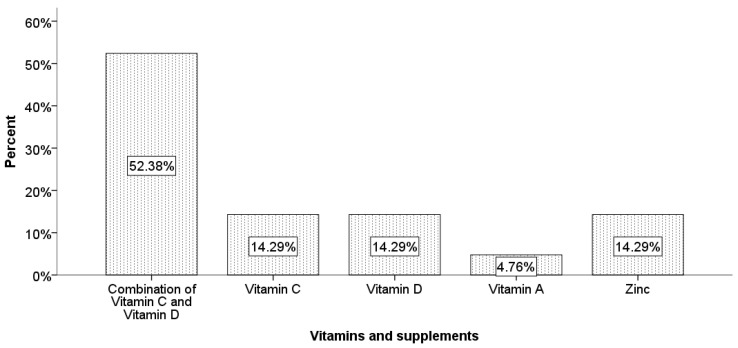
Percentage distribution of used vitamins and supplements.

During the coronavirus pandemic, 33.96% of respondents stated that they used vitamins as part of self-medication, and 5.66% used supplements, which is 39.62% in total. Of the supplements, all 5.66% reported using zinc. Of the vitamins, respondents mostly used a combination of vitamins C and D (11–20.75%), vitamin D (3–5.66%), vitamin C (3–5.66%), and vitamin A (1–1.89%). If we consider only vitamins and supplements, their percentage distribution is shown in Figure 1.

Descriptive statistical analysis related to respondents’ self-medication was also performed using absolute and relative frequencies and is shown in Table 3.

Analysis of socio-demographic characteristics and characteristics related to the health status of respondents in relation to the application, recommendation, and effect of self-medication during the pandemic is shown in Table 4.

In order to determine which of the respondents’ socio-demographic characteristics has the greatest influence on the use of self-medication, we applied binary logistic regression. The applied prediction model explains between 15% and 20.2% of the total variance of the dependent variable. Given that the chi-square indicator value for the Hosmer and Lemeshow test is 5.567 with a significance of 0.696, which is greater than 0.05, this model is considered good. Based on the significance values from Table 5, we see that only unemployment and vaccination have a significant impact on the use of self-medication. Unemployment has a negative effect, while vaccination has a positive effect. Given that the value of the Wald coefficient is higher for vaccination, we conclude that vaccination has the greatest unique and statistically significant influence on the use of self-medication. If a person is vaccinated, it means that there is a 3.756 times greater probability that they will self-medicate.

## 4. Discussion

According to previous research, the most common reasons for self-medication were saving money and time, mild symptoms of illness, and previous experience with self-medication [18]. In our research, the process of self-medication during the COVID-19 pandemic was applied by 73.1% of respondents. This percentage is higher compared to the average reflected in other countries, but it is certainly within the expected limits. During the coronavirus pandemic, the self-medication process ranged from 4% to 88.3%, depending on the region [9]. Regarding the region, the highest prevalence of self-medication was in Asia (53%), while the lowest was in Europe (40.8%). In terms of population, the highest prevalence was among students (54.5%) and the lowest among healthcare workers (32.5%) [19]. In a large number of studies, the percentage of self-medication ranged from 40 to 60% [3,15,20]. The prevalence of self-medication in the general population was 48.8% [19].

As we can see in Table 1, our investigation included more female participants than male. This is important to consider when explaining the influence of gender on COVID-19. Gender played a significant role in COVID-19 outcomes, with men generally facing more severe disease and higher mortality rates, while women may have stronger immune responses but are more prone to long-term effects. These patterns are influenced by a range of factors, including biological, behavioral, and social determinants. Therefore, gender cannot be viewed as an isolated factor contributing to COVID-19 disease and outcomes.

If we observed the employment status in our study, we can see that more than half of the participants were employed. Employment status can significantly influence how individuals approach their health during COVID-19, including their use of OTC medications. Factors such as financial stability, healthcare access, workplace exposure, and stress levels all played roles in shaping decisions around self-medication. Besides employment status, monthly income can significantly affect OTC use. As mentioned in the results, our subjects had monthly incomes ranging from EUR 350 to 680, which is not a large amount and could negatively influence OTC use.

The prevalence of self-medication in the case of treatment for the coronavirus is significantly lower, and in our research, it is 26.9%. In addition, in the works of other researchers, this percentage is lower compared to the prevalence of self-medication as a form of prevention and ranges from 7.4% to 41.7%. The prevalence of self-medication among patients with coronavirus was 41.7% [19]. During the coronavirus pandemic, the prevalence of self-medication increased by 3.2% from 2020 to 2021 [21]. The increase in the prevalence of self-medication was from 48% in 2020 to 51.2% in 2021 [19,21]. Some researchers state that the prevalence of self-medication decreased after the end of the pandemic, as it was 88% before the pandemic and 57% after the pandemic [22]. According to the results of our research, self-medication as a form of prevention and treatment is used more by women in 61.71% of cases, which is in agreement with other research in which it was found that this percentage is in the range of 53.4% [3] to 58.2% [21]. The most common reasons for self-medication were headache (45%), menstrual pain (23%), and fever (14%) [4]. In addition to the prevention and treatment of COVID-19 [23], the self-medication process was also used for fever, cough, body aches, sore throat, and headaches [18]. In our research, we came to the conclusion that of the drugs that are taken without a prescription, analgesics are most often used in 52.83% of cases, which is in agreement with the results of other researchers who came to the conclusion that the most commonly used types of drugs were analgesics, antibiotics, and supplements [15]. The most commonly used types of drugs are antibiotics, hydroxychloroquine, acetaminophen, vitamins and supplements, ivermectin, and ibuprofen [9]. The most commonly used medications in the self-medication process were antibiotics, herbal-based medicines, vitamins, and analgesics [18], while the medications most frequently used for the treatment of COVID-19 were hydroxychloroquine, azithromycin, ivermectin, etc. [24]. These medications showed promising results in in vitro and small-scale studies, but these results were not confirmed in large randomized controlled trials [23]. Of the supplements, respondents used zinc, and of the vitamins, most often they used a combination of vitamins C and D, followed by vitamin D, vitamin C, and vitamin A.

In the largest number of cases (41.1%), the respondents received advice on the use of self-medication from their chosen doctor, followed by 36% of cases from a pharmacist and least of all from family and friends. The information on self-medication before conducting the investigation was most commonly obtained from family and friends, social media, and healthcare workers [18]. According to their opinion, less than half of the respondents (41.1%) believe that the process of self-medication had an effect.

Self-medication without proper guidance can lead to the incorrect use of drugs, incorrect dosage, or adverse drug reactions. Research shows that patients who consult healthcare professionals are less likely to misuse medications or experience harmful side effects. Studies have highlighted that pharmacists and doctors can provide crucial advice about potential drug interactions, ensuring the safety of the patient and avoiding dangerous combinations. Poor self-medication practices contribute to larger public health issues, such as antibiotic resistance and the misuse of over-the-counter drugs. By seeking advice from a doctor or pharmacist, individuals are more likely to follow evidence-based practices, reducing the broader health risks posed by incorrect self-medication. During the COVID-19 pandemic, the importance of seeking advice on the use of self-medication from a chosen doctor or pharmacist became even more critical due to the uncertainty surrounding the virus and its treatments and the increased reliance on self-medication as a result of lockdowns and overwhelmed healthcare systems [18]. Compared to the aforementioned information and the results from our study (41.1% of participants take recommendations for self-medication from their chosen physician and 36% from a pharmacist), we can see that study participants showed good habits regarding the sources of information for self-medication. This attitude can create favorable conditions for self-medication in future epidemic situations.

The process of self-medication is related to the respondent’s age, employment status, marital status, personal income, visits to the doctor, chronic diseases, and vaccination status in the research we conducted. Self-medication for the treatment of COVID-19 was used at a significantly higher percentage by younger respondents (M = 39.49 years old), respondents who are not married, respondents without their own income, respondents who rarely visit a doctor (once a year or less), respondents who do not have chronic diseases, and unvaccinated subjects. The factors influencing the decision to self-medicate were primarily fear of infection and limited access to healthcare facilities. The most significant factors affecting their decision to self-medicate were the severity of the illness, gender, age, education level, and marital status [18,23]. As we can see, the results we obtained are consistent with findings from studies conducted on this topic in other countries.

The recommendation for self-medication in our research was related to the respondent’s age, work status, marital status, personal income, chronic diseases, trust in the chosen doctor and pharmacist, positive diagnosis for COVID-19, and vaccination status. A significantly higher percentage of recommendations for self-medication from the chosen doctor is received by older respondents, pensioners, married respondents, those with personal income, respondents with chronic diseases, respondents who have tested positive for COVID-19, and those who have been vaccinated. The correlation between recommendations for self-medication from the chosen doctor and vaccination in our subjects can be explained by the fact that vaccinated individuals have already made an active decision to follow public health recommendations (i.e., getting vaccinated), which suggests they are more likely to follow medical advice responsibly.

The effect of self-medication was related to the age of the subjects, employment status, personal income, visits to the doctor, trust in the doctor, and a positive COVID-19 diagnosis. The effect of self-medication was significantly more pronounced in younger subjects, the unemployed, those without personal income, individuals who visit the doctor less often, those who have confidence in their chosen doctor, and subjects who did not test positive for COVID-19.

Vaccination has the greatest unique and statistically significant influence on the use of self-medication. If a person is vaccinated, there is a 3.756 times higher probability that they will use self-medication.

For the effective implementation of the self-medication process, a holistic approach is necessary. This approach should include public education, training by healthcare professionals, active monitoring of medication sales in pharmacies, and stricter regulation of public advertising. To prevent the spread of misinformation on social media and the internet in general, text mining should be applied to detect such information [24].

On the other hand, there are some limitations to this study that must be pointed out. The first limitation is the use of a self-reported questionnaire in this research. Another limitation is that the study was conducted at only one health center, and the study sample was consecutive rather than randomized. Additionally, the study sample consisted of more female participants than male. Furthermore, there was no control group for this study. Apart from these points, participants were not analyzed by age group. These limitations could be addressed through further qualitative research to obtain a broader and deeper understanding of the examined questionnaire. Due to these limitations, readers should interpret our results with caution.

## 5. Conclusions

During the coronavirus pandemic, the process of self-medication became an integrated part of the healthcare approach. The respondents maintained a high level of trust in their chosen doctor and pharmacist, from whom they mostly received advice on self-medication. This is in favor of the proper application of self-medication, as indicated by the high level of vaccination.

Elderly respondents, pensioners, those who are married, have personal income, have chronic diseases, or were both positive for COVID-19 and vaccinated, mostly received advice on self-medication from a doctor. Self-medication for the treatment of COVID-19 was more often used by younger respondents and those who are not yet married, do not have their own income, and rarely visit a doctor. Among these respondents, self-medication had a significantly greater effect.

Zinc was the most commonly used supplement, while a combination of vitamins C and D was the most frequently used when it came to vitamins. Vaccination had the greatest unique and statistically significant influence on the use of self-medication. If a person is vaccinated, there is a 3.756 times higher probability that they will use self-medication.

By understanding the relationship between vaccination status, self-medication behaviors, and the use of supplements, policymakers can develop targeted interventions to promote safe self-medication, prevent misuse, and ensure that individuals make well-informed health decisions. These interventions could include public health campaigns, regulation of supplement sales, improved access to healthcare services, and clear guidelines on when and how self-medication is appropriate.

## Figures and Tables

**Table 1:** Demographic and social characteristics of the participants.

Variable	Answer	Number of Respondents	Percentage of Respondents
Gender of the respondent	Female	108	61.71%
	Male	67	38.29%
Level of education	Primary and secondary school	87	49.71%
	Higher school and college	40	22.86%
	Faculty	48	27.43%
Employment status	Employed	104	59.43%
	Unemployed	26	14.86%
	Pensioner	45	25.71%
Marital status	Single	27	15.43%
	In a relationship	26	14.86%
	Married	122	69.71%
Personal monthly income	He/she has	149	85.14%
	He/she does not have	26	14.86%
Monthly income of the family	<40,000	40	22.86%
	40,000–80,000	88	50.29%
	>80,000	47	26.86%
Place of living	Village	35	20.00%
	City	140	80.00%
Medical education in the family	Yes	62	35.43%
	No	113	64.57%
Do you know someone who died as a result of the COVID-19 pandemic?	Yes	147	84.00%
	No	28	16.00%

**Table 2:** Descriptive statistical analysis related to the health status of the respondents.

**Variable**	**Answer**	**Number of Respondents**	**Percentage of Respondents**
Visit to the doctor	Once a month	29	16.57%
	Once every six months	69	39.43%
	Once a year	42	24.00%
	Rarer than the above	35	20.00%
Chronic diseases	Yes	83	47.43%
	No	92	52.57%
Which chronic diseases	Hypertension	52	62.65%
	Endocrine diseases	15	18.07%
	Other diseases	16	19.28%
Buying prescription drugs	Yes	79	95.18%
	No	4	4.82%
Confidence in the chosen doctor	Yes	168	96.00%
	No	7	4.00%
Trust in the chosen pharmacist	Yes	162	92.57%
	No	13	7.43%
Buying medicines without a prescription	Yes	94	53.71%
	No	81	46.29%
Which drugs	Analgesics	28	52.83%
	Vitamins	18	33.96%
	Probiotics	4	7.55%
	Supplements	3	5.66%
Positive for COVID-19	Yes	106	60.57%
	No	69	39.43%
How many times	Once	77	72.64%
	Twice and more	29	28.36%
Place of treatment	Community health center	91	85.85%
	Hospitalization	15	14.15%
Vaccination against COVID-19(fully vaccinated)	Yes	130	74.29%
	No	45	25.71%

**Table 3:** Descriptive statistical analysis related to the respondents’ self-medication.

Variable	Answer	Number of Respondents	Percentage of Respondents
Self-medication during the pandemic	Just as a prevention	128	73.1%
	For the treatment of COVID-19	47	26.9%
Recommendation for self-medication	Chosen physician	72	41.1%
	Pharmacist	63	36.0%
	Family and friends	40	22.9%
Improvement of clinical status	Yes	72	41.1%
	No	103	58.9%

**Table 4:** Analysis of socio-demographic characteristics and characteristics related to the health status of respondents in relation to the application, recommendation, and effect of self-medication during the pandemic.

	Self-Medication		Recommendation		Effect	
	Stat.	R	Stat.	R	Stat.	R
Gender of the respondent	3.071	0.080	4.725	0.094	2.082	0.149
Age of the respondents	t = 8.215	0.000	t = 8.743	0.000	t = 4.018	0.000
Level of education	2.341	0.310	8.438	0.077	5.580	0.061
Employment status	19.189	0.000	54.795	0.000	21.289	0.000
Marital status	6.421	0.040	12.454	0.014	5.887	0.053
Personal incomes	11.323	0.001	16.430	0.000	12.860	0.000
Family incomes	0.845	0.655	3.156	0.532	0.405	0.817
Place of living	0.029	0.865	2.091	0.352	2.855	0.091
Medical education	0.015	0.901	5.801	0.055	3.111	0.078
Death from COVID-19	0.500	0.479	0.406	0.816	0.406	0.524
Visit to the doctor	17.251	0.001	10.759	0.096	10.615	0.014
Chronic diseases	17.626	0.000	21.615	0.000	2.509	0.113
Trusting the doctor	0.587	0.444	16.559	0.000	5.097	0.024
Trust in the pharmacist	0.109	0.741	8.272	0.016	0.042	0.838
Self-medication (before)	0.007	0.933	5.161	0.076	1.281	0.258
Positive diagnosis	0.742	0.389	10.670	0.005	9.128	0.003
Place of treatment	3.011	0.083	0.894	0.640	2.817	0.093
Vaccination	7.280	0.007	27.602	0.000	0.763	0.382

**Table 5:** Examination of the influence of certain factors on self-medication during the COVID-19 pandemic.

Variables	B	SE	Wald	Df	P	OR	95% CI	
							Lower	Upper
Age	−0.020	0.014	2.017	1	0.156	0.980	0.953	1.008
Unemployment	−1.523	0.601	6.426	1	0.011	0.218	0.067	0.708
Marriage	0.608	0.378	2.578	1	0.108	1.836	0.875	3.855
Monthly visit to the doctor	0.417	0.458	0.827	1	0.363	1.517	0.618	3.727
Chronic diseases	0.614	0.403	2.318	1	0.128	1.848	0.838	4.076
Vaccination	1.323	0.444	8.890	1	0.003	3.756	1.574	8.966
Constant	−0.887	0.752	1.391	1	0.238	0.412		

## Data Availability

Data available on request due to restrictions (e.g., privacy, legal or ethical reasons).
